# Functional cross-talk between phosphorylation and disease-causing mutations in the cardiac sodium channel Na_v_1.5

**DOI:** 10.1073/pnas.2025320118

**Published:** 2021-08-09

**Authors:** Iacopo Galleano, Hendrik Harms, Koushik Choudhury, Keith Khoo, Lucie Delemotte, Stephan Alexander Pless

**Affiliations:** ^a^Department of Drug Design and Pharmacology, University of Copenhagen, Copenhagen 2100, Denmark;; ^b^Science for Life Laboratory, Department of Applied Physics, KTH Royal Institute of Technology, Solna SE-171 65, Sweden

**Keywords:** protein engineering, cardiac arrhythmia, pharmacology, sodium channel inactivation, personalized medicine

## Abstract

The voltage-gated sodium channel Na_v_1.5 initiates the cardiac action potential. Alterations of its activation and inactivation properties due to mutations can cause severe, life-threatening arrhythmias. Yet despite intensive research efforts, many functional aspects of this cardiac channel remain poorly understood. For instance, Na_v_1.5 undergoes extensive posttranslational modification in vivo, but the functional significance of these modifications is largely unexplored, especially under pathological conditions. This is because most conventional approaches are unable to insert metabolically stable posttranslational modification mimics, thus preventing a precise elucidation of the contribution by these modifications to channel function. Here, we overcome this limitation by using protein semisynthesis of Na_v_1.5 in live cells and carry out complementary molecular dynamics simulations. We introduce metabolically stable phosphorylation mimics on both wild-type (WT) and two pathogenic long-QT mutant channel backgrounds and decipher functional and pharmacological effects with unique precision. We elucidate the mechanism by which phosphorylation of Y1495 impairs steady-state inactivation in WT Na_v_1.5. Surprisingly, we find that while the Q1476R patient mutation does not affect inactivation on its own, it enhances the impairment of steady-state inactivation caused by phosphorylation of Y1495 through enhanced unbinding of the inactivation particle. We also show that both phosphorylation and patient mutations can impact Na_v_1.5 sensitivity toward the clinically used antiarrhythmic drugs quinidine and ranolazine, but not flecainide. The data highlight that functional effects of Na_v_1.5 phosphorylation can be dramatically amplified by patient mutations. Our work is thus likely to have implications for the interpretation of mutational phenotypes and the design of future drug regimens.

## Significance

The cardiac sodium channel (Na_v_1.5) is crucial for generating a regular heartbeat. It is thus not surprising that Na_v_1.5 mutations have been linked to life-threatening arrhythmias. Interestingly, Na_v_1.5 activity can also be altered by posttranslational modifications, such as tyrosine phosphorylation. Our combination of protein engineering and molecular modeling has revealed that the detrimental effect of a long QT3 patient mutation is only exposed when a proximal tyrosine is phosphorylated. This suggests a dynamic cross-talk between the genetic mutation and a neighboring phosphorylation, a phenomenon that could be important in other classes of proteins. Additionally, we show that phosphorylation can affect the channel’s sensitivity toward clinically relevant drugs, a finding that may prove important when devising patient-specific treatment plans.

The cardiac action potential is elicited by the temporally precisely orchestrated activity of voltage-gated sodium, potassium, and calcium channels. The voltage-gated sodium ion channel Na_v_1.5, for which more than 500 potentially pathologically relevant point mutations have been reported (1), is responsible for the initial fast depolarization observed in cardiac action potentials. Many of these mutations are the known or suspected cause of aberrant action potentials that can result in life-threatening conditions like Brugada and long QT3 syndromes (BS and LQT3, respectively) (2). The most common noninvasive treatment for such conditions includes administration of antiarrhythmic drugs (AADs), such as quinidine, flecainide, or ranolazine (3).

Na_v_1.5 is a 2,016-amino-acid membrane protein with four homologous but distinct domains (DI-IV) (4). Each domain contains six transmembrane segments (S1 to S6). The first four segments (S1 to S4) form the so-called “voltage sensing domain,” in which the positively charged residues of S4 are responsible for the voltage sensitivity of the channel. Conversely, the last two segments (S5 and S6), and the P-loop that connects them, form the pore module, including the selectivity filter. Upon depolarization, the S4 segments undergo an upward movement, which is translated to conformational changes that open the channel gate. This channel opening is primarily mediated by S4 helices in DI-DIII, while the slower upward movement of DIV causes the channel to inactivate (5–8). The latter is caused by the isoleucine, phenylalanine, and methionine (IFM) tripeptide motif in the DIII-DIV linker binding to its receptor site adjacent to DIV S6 (9–13). Subthreshold depolarizations can cause the channel to undergo steady-state inactivation (SSI) without prior opening. The voltage dependence of SSI is an important determinant of channel availability in vivo, and even small alterations can give rise to arrhythmic phenotypes. Similarly, incomplete inactivation can result in a potentially pathogenic standing current referred to as a late current (14).

Intriguingly, many of the known or suspected disease-causing mutations occur in the cytosolic interdomain linkers and the loops between individual transmembrane segments (1, 11, 15–17). These loops and linkers are not only hotspots for disease-causing mutations but also contain numerous posttranslational modifications (PTMs), such as phosphorylation, methylation, and acetylation (18). Phosphorylation stoichiometries are typically highly variable (19), and previous work has shown that phosphorylation levels can increase substantially in cardiac ion channels, such as Na_v_1.5 and K_v_7.1, upon β-adrenergic stimulation, for example (20). Consequently, mutations at PTM sites or dysregulation of PTM levels play important roles in disease states (18, 21). Yet despite firm evidence for their presence and relevance in vivo, it has remained challenging to directly assess the functional effects of PTMs in vitro: conventional mutagenesis can be used to introduce nonmodifiable (NM) side chains to prevent modification, but mimicking PTMs using naturally occurring side chains is often flawed, especially with mimicking phosphorylation of tyrosine (22). Similarly, overexpression of regulatory enzymes (e.g., kinases or phosphatases) lacks selectivity toward the protein of interest. Recently, we overcame some of these limitations by using tandem protein trans-splicing (tPTS) to generate semisynthetic membrane proteins containing stable PTM mimics (23).

Here, we employ a combination of tPTS, electrophysiology, and molecular dynamics (MD) simulations to investigate if the functional effects caused by phosphorylation of Na_v_1.5 Y1495 (10 mV right-shift in the V_1/2_ of SSI) (23, 24) are altered by clinically relevant mutations in its proximity and if they affect the pharmacological profile of AADs in wild-type (WT) and mutant channels. Our data show that in contrast to the pathogenic ΔK1500 mutant (25), the disease-causing Q1476R mutation (26) has no significant impact on SSI per se. However, when the Q1476R mutation is combined with phosphorylation at the nearby Y1495, this leads to a dramatically right-shifted SSI (20 mV) and a prominent late current. Our MD simulations attribute these shifts in SSI to less stable binding of the IFM inactivation particle in channels with phosphorylation at 1495, in particular when combined with the Q1476R mutation. Finally, we show that Na_v_1.5 sensitivity toward two class I AADs and ranolazine is altered by phosphorylation and the tested patient mutations.

## Results

### Experimental Strategy.

**Fig. 1.**
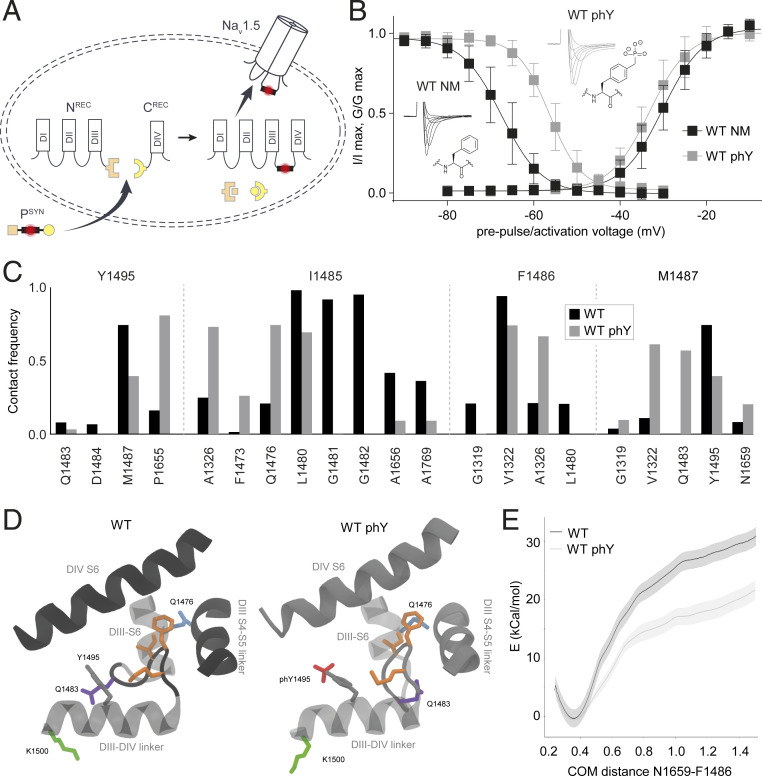
Phosphorylation of Y1495 destabilizes docking of IFM motif into its receptor site. (*A*) Schematic of tPTS used to generate Na_v_1.5 channels that are NM (WT NM) or phosphorylated (WT phY) at the Y1495 position. Amino acids (aa) 1 to 101 of *Cfa*DnaE (orange) are merged to the C terminus of channel fragment corresponding to Na_v_1.5 aa 1 to 1471 for heterologous expression as the N^REC^ construct. The P^SYN^ sequence corresponds to Na_v_1.5 aa 1472 to 1502 and is linked to the C-terminal part of *Cfa*DnaE (aa 102 to 137, orange) at its N terminus and the N-terminal part of *Ssp*DnaB^M86^ (aa 1 to 11, yellow) at its C terminus. The corresponding C-terminal part of *Ssp*DnaB^M86^ (aa 12 to 154, yellow) is expressed as a fusion construct at the N terminus of protein fragment C (Na_v_1.5 aa 1503 to 2016) to form the C^REC^ construct. (*B*) SSI (*Left*) and activation (*Right*) curves of WT NM and WT phY constructs, including example traces and chemical structure of the aa present in position 1495. Data shown as mean ± SD; *n* = 6 to 9. (*C*) Contact frequency of Y1495 and IFM particle residues with neighboring residues. Phosphorylation reduces the contact between Y1495 and M1487 of IFM, while it causes the IFM particle to increase contacts with DIII-S6 and DIII–S4-S5 linker. (*D*) Conformation of IFM particle in its binding site after 200 ns of MD simulation. Phosphorylation of Y1495 moves the loop region of the DIII-DIV linker containing the Q1483 residue outward and the IFM particle closer to the DIII-S6. IFM side chains are highlighted in orange. (*E*) Free energy profile of IFM unbinding, using the distance between the center of mass (COM) of N1659 in the DIV-S5 helix and F1486 in the IFM particle as a reaction coordinate. WT phY causes a decrease in the binding energy of IFM binding.

Heterologous protein expression does not allow precise control over the extent of PTM. In the case of Na_v_1.5 Y1495, while a conventional mutagenesis approach can prevent phosphorylation with a tyrosine-to-phenylalanine mutation, it is not possible to control the degree of phosphorylation on the native tyrosine. We overcome this obstacle using a recently developed semisynthetic approach that allows for the insertion of synthetic peptides carrying single or multiple PTMs or PTM mimics into ion channels (23). In this approach, the channel is divided into three fragments: N- and C-terminal fragments (N^REC^ and C^REC^) corresponding to partial channel fragments that are recombinantly expressed in *Xenopus laevis* oocytes and a synthetic peptide (P^SYN^) containing the site of interest, which is injected into the oocyte cytosol (Fig. 1*A* and *SI Appendix*, Fig. S1). The covalent linkage of the three fragments is mediated by two orthogonal split inteins: *Cfa*DnaE (split intein A) (27) and *Ssp*DnaB^M86^ (split inteins B) (28). Upon assembly, the respective split inteins spontaneously and covalently link the attached channel fragments via native chemical ligation, a process also termed tPTS (29) (Fig. 1*A* and *SI Appendix*, Figs. S1 and S2).

This approach allows us to insert P^SYN^ variants either containing a phenylalanine (which cannot be posttranslationally modified and is hence designated NM) or a phosphonylated tyrosine (30) in position 1495 (phY). The latter is a hydrolytically stable phosphorylation mimic with near identical charge and steric properties (31). We will thus refer to effects of phosphorylation throughout the manuscript whenever we introduce phY. To increase splicing efficiency, we introduced the N1472C mutation at the first position of P^SYN^, which results in a right-shift in SSI compared to WT channels (23) (Table 1). This is consistent with the notion that the highly similar N1472S variant has previously been reported as a putative long QT-associated mutation (32).

**Table 1.** Electrophysiological characterization

Construct	V_50_ activation (mV)	V_50_ SSI (mV)	I _late_ (% of I _peak_)	τ _inactivation_ (ms)	τ _recovery_ (ms)
WT	−37.3 ± 2.3 (9)	−76.7 ± 1.9 (9)	nd	nd	nd
WTrec	−34.4 ± 3.1 (7)	−67.7 ± 3.5 (8)	nd	nd	nd
WT NM	−30.1 ± 2.9 (7)	−67.5 ± 3.6 (7)	−4.6 ± 2.7 (6)	1.3 ± 0.3 (7)	20.9 ± 4.3 (7)
WT phY	−32.8 ± 3.2 (7)	−56.4 ± 2.2 (6)	−2.4 ± 3.2 (7)	1.7 ± 0.3 (7)	9.3 ± 1.5 (5)
Q1476R NM	−38.3 ± 2.5 (7)	−68.1 ± 2.5 (7)	−2.2 ± 1.9 (10)	1.8 ± 0.2 (6)	14.8 ± 1.5 (6)
Q1476R phY	−33.2 ± 6.7 (6)	−47.8 ± 5.0 (6)	10.7 ± 4.5 (5)	2.9 ± 0.7 (8)	6.7 ± 1.4 (7)
ΔK1500 NM	−29.1 ± 2.6 (7)	−71.3 ± 3.4 (6)	14.5 ± 2.8 (6)	3.0 ± 0.5 (6)	nd
ΔK1500 phY	−33.0 ± 3.5 (6)	−59.4 ± 3.2 (7)	9.9 ± 5.4 (10)	3.1 ± 0.8 (6)	nd
Y1495E	−30.0 ± 2.0 (10)	−55.8 ± 2.1 (11)	nd	nd	nd
Q1476R+Y1495E	−30.9 ± 1.5 (6)	−50.7 ± 2.3 (10)	nd	nd	nd

Values for half-maximal activation (V_50_ activation, in millivolts) and SSI (V_50_ SSI, in millivolts) are listed, along with late currents observed 400 ms after the peak current elicited by a voltage step to −30 mV [I _late_ (% of I _peak_)] and the time constant for inactivation in response to a voltage step to −20 mV (τ _inactivation_, in milliseconds) as well as the time constant for recovery from inactivation in response to 2 voltage steps to −20 mV with varying recovery intervals at −80 mV in between (τ _recovery_, in milliseconds). Data are provided as mean ± SD; the number of replicates for each value is shown in parentheses; nd, not determined.

### Phosphorylation of Y1495 Alters Interaction between IFM Tripeptide and Receptor Site.

Injection of oocytes expressing N and C fragments with NM peptide (WT NM) results in activation and SSI parameters similar to those obtained when all three channel fragments are expressed recombinantly (WT^rec^) (Table 1 and *SI Appendix*, Fig. S3). However, no voltage-dependent currents were observed within similar incubations times when only N and C fragments were injected, suggesting that the channel fragments alone were unable to noncovalently assemble (23). Consistent with previous work, phosphonylation at Y1495 (WT phY) results in a roughly 10-mV right-shift in the SSI curve while retaining WT-like values for half-maximal activation (Fig. 1*B* and Table 1) (23, 33). The time course of inactivation remained unchanged and no late current was observed, while the time course of recovery from inactivation of WT phY was significantly accelerated relative to the one of WT NM (Table 1 and *SI Appendix*, Fig. S4).

To elucidate the molecular basis for these experimental observations, we turned to MD simulations of the recently determined cryogenic electron microscopy structure of Na_v_1.5 in which the IFM particle is docked to a site between the DIII S4-S5 linker and DIV S6 (11). Since SSI is attributed to this specific interaction (Fig. 1*D*), we focused on differences between WT and WT phY in this region. Strikingly, 200 ns MD simulations reveal that phosphorylation causes a loss in contact of Y1495 with M1487 of the IFM particle. In WT phY, a contact between I1485 of IFM and F1473 and Q1476 (in DIII-S6) appears (I1485, Fig. 1*C*). Interactions involving F1486, on the other hand, display no substantial differences between these two conditions, except for the formation of contacts with A1326 upon phosphorylation (F1486, Fig. 1*C*). The phosphate group of WT phY mediates a downward movement of Q1483 and moves D1484 away from DIV-S6 (Y1495, Fig. 1 *C* and *D*). Thus, upon phosphorylation of Y1495, the phosphate group moves toward the binding pocket of the IFM particle (Fig. 1*D*), pushing the IFM particle toward DIII-S6 and forming contacts with Q1476. This causes the N-terminal part of the DIII-DIV linker (Q1483, D1484) to move out and downward, away from the IFM particle binding pocket (Fig. 1*D*).

Umbrella sampling simulations were carried out to evaluate the binding free energy of the IFM particle to its docking site, using the distance between the center of mass (COM) of N1659 in DIV-S5 helix and F1486 in the IFM particle as a reaction coordinate (Fig. 1*E*). This specific reaction coordinate was chosen because N1659 forms a hydrogen bond with the carbonyl group of F1486 in the docked state, as suggested by Jiang et al. (11). As expected from the observations from regular MD simulations, phosphorylation of Y1495 indeed causes the binding free energy to decrease, suggesting that the unbinding of the IFM particle is eased by phosphorylation. This is in good agreement with the right-shift in SSI and the accelerated recovery from inactivation observed experimentally.

Together, the combined experimental and computational data support the notion that phosphorylation at Y1495 destabilizes the interaction of the IFM motif with its receptor site. Specifically, phosphorylation of Y1495 moves it away from the DIV-S6 and toward the DIII-S6 and DIII-S4-S5 linker, thereby right-shifting Na_v_1.5 SSI and accelerating recovery from inactivation.

### Q1476R Pathogenicity Likely Arises from Phosphorylation at Y1495, Not the Mutation Per Se.

**Fig. 2.**
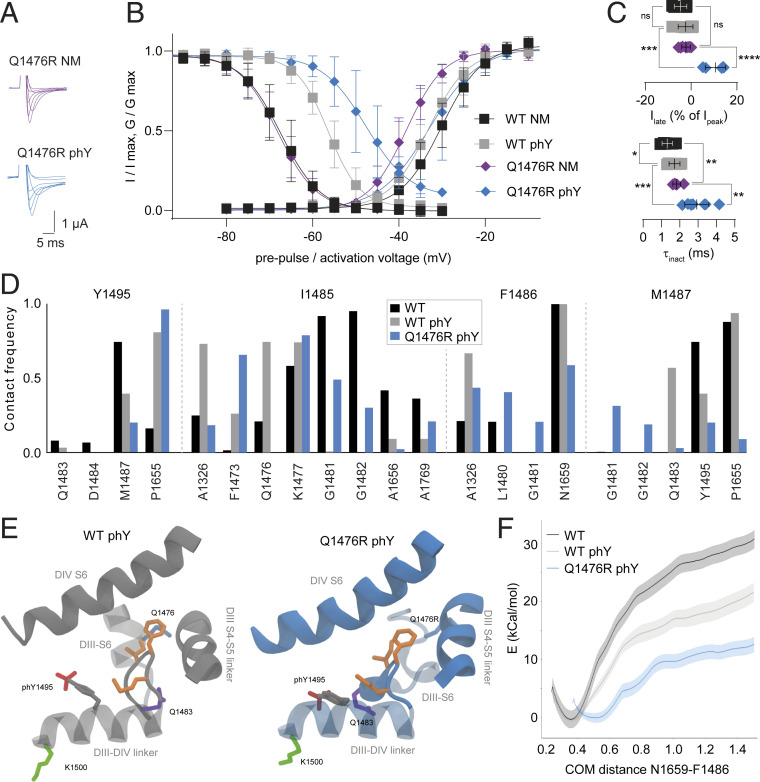
The Q1476R mutation results in a dramatically shifted SSI. (*A*) Representative current traces for Q1476R NM (purple) and phY (blue) constructs. (*B*) SSI (*Left*) and activation (*Right*) curves of indicated constructs. (*C*) Late currents (*Upper*) and inactivation rates (*Lower*) for indicated constructs. Data are shown as mean ± SD in *B* and *C*; *n* = 5 to 10; data were compared using unpaired two-tailed Student’s *t* test; ns (not significant) *P* > 0.05, *P ≤ 0.05, **P ≤ 0.01, ***P ≤ 0.001, and ****P < 0.0001. Note that the precise measurements of late currents in *X. laevis* oocytes are hampered by slow-onset, voltage-dependent endogenous currents. Therefore, our late current measurements can only serve as an estimate. (*D*) Contact frequency of Y1495 and IFM particle with neighboring residues. (*E*) Conformation of IFM particle in its binding site after 200 ns of MD simulation. Q1476R phY leads to a break in the DIII-S6 helix, leading to the destabilization of IFM binding. IFM side chains are highlighted in orange. (*F*) Free energy profile of IFM unbinding, using the distance between the COM of N1659 in DIV-S5 helix and F1486 in the IFM particle as a reaction coordinate. Q1476R phY results in a further decrease of binding energy of the IFM particle compared to WT phY and an increased equilibrium distance between F1486 and its docking site due to the break in DIII-S6 induced by the Q1476R mutation.

The Q1476R mutation in the DIII-DIV linker had previously been suggested to be the cause of LQT3 in a patient family (26). Specifically, heterologous expression in mammalian cells had demonstrated that the mutant channels displayed a 6.5-mV right-shift in SSI compared to WT, along with the emergence of a late current (26). Given the close physical proximity of Q1476 and Y1495 with the IFM inactivation particle and the increased contact frequency of I1485 and Q1476 observed in WT phY, we sought to test if phosphorylation of Y1495 would have a distinct effect on channel function in the presence of the Q1476R mutation. To this end, we synthesized P^SYN^ variants containing the Q1476R mutation on either the Y1495F (Q1476R NM) or Y1495 phY background (Q1476R phY). Insertion of either peptide variant resulted in robust voltage-gated currents within 12 h after injection (Fig. 2*A*). To our surprise, introducing the Q1476R mutation on the nonphosphorylated background did not shift the SSI voltage dependence or elicit a late current (Fig. 2 *B* and *C* and Table 1). By contrast, when we introduced the Q1476R mutation in the presence of a phosphorylated tyrosine at 1495, we observed a drastic right-shift in SSI compared to Q1476R NM (ΔSSI ∼20 mV, Table 1), significantly beyond what we observed on the WT background (ΔSSI ∼11 mV). We further show that phosphorylation on the mutant (but not the WT) background also significantly slowed the rate of channel inactivation and resulted in a pronounced late current after 400 ms (Fig. 2 *A* and *C* and Table 1).

The only parameter that was significantly affected by Q1476R alone (Q1476R NM) was the voltage required for half-maximal activation. This was significantly left-shifted compared with WT NM but indistinguishable for Q1476R phY and WT phY (Fig. 2 *B* and *C* and Table 1).

These data suggest that the phosphorylation-induced destabilization of the IFM particle in its receptor site was starkly exacerbated in the presence of the pathogenic Q1476R mutation. This idea is further supported by our finding that recovery from inactivation was accelerated for Q1476R phY compared with WT phY (Table 1 and *SI Appendix*, Fig. S4).

Next, we sought to assess if full-length Na_v_1.5 containing a conventional glutamic acid as a mimic of phosphotyrosine in position 1495 on either the WT or the Q1476R background would yield comparable results (Y1495E and Q1476R/Y1495E, respectively). Although we observed overall similar trends, both Y1495E and Q1476R/Y1495E resulted in even more pronounced shifts in SSI compared to the semisynthetic WT NM, WT phY, and Q1476R phY constructs (Table 1 and *SI Appendix*, Fig. S3). This underscores the advantages of inserting phosphorylation mimics with near identical charge and steric properties via our semisynthetic approach rather than using conventional mutagenesis.

To rationalize the molecular basis for the observed effect caused by Q1476R, we conducted 200-ns-long MD simulations of Na_v_1.5 with both the WT phY and the Q1476R phY systems. Strikingly, the Q1476R mutation causes a break in the DIII-S6 helix, allowing its C-terminal end to move into the IFM binding site. As a consequence, increased contact frequency was observed between G1481/G1482 and the IFM motif in the Q1476R phY system compared to the WT phY system (Fig. 2 *D* and *E*). The Q1476R mutation also leads to a loss of contact of position 1476 itself to I1485, suggestive of the importance of this contact for stabilizing the IFM motif in the WT phY system. We also observe a decrease in contact frequency between F1486 of IFM and N1659 of DIV-S6 and the formation of new contacts with L1480 and G1481 (Fig. 2 *D* and *E*). Finally, M1487 reduces its interaction with P1655, Q1483, and phY in position 1495 while increasing its contact with G1481 and G1482. Our umbrella sampling results also indicate that the combination of Q1476R and phY further decreases the binding free energy of the IFM motif to its binding site and leads to a larger equilibrium distance between the F1486 and its docking site, in line with expectations from regular MD simulations (Fig. 2*F*).

Overall, we show that the effects of Y1495 phosphorylation on SSI are strongly enhanced in the presence of the Q1476R patient mutation, while the latter has little effect in the absence of the Y1495 phY modification. In WT phY, we observe increased contacts of IFM with DIII-S6, in particular through contacts between I1485 and Q1476. Thus, mutating Q1476 to R appears to further destabilize the IFM interactions with its docking site in a phosphorylated state. Together, this implies that the pathogenicity of the Q1476R mutation does not arise from the amino acid change per se but rather the functional effects conferred by a nearby phosphorylation.

### Phosphorylation at Y1495 Has Similar Effects on ΔK1500 and WT Channels.

**Fig. 3.**
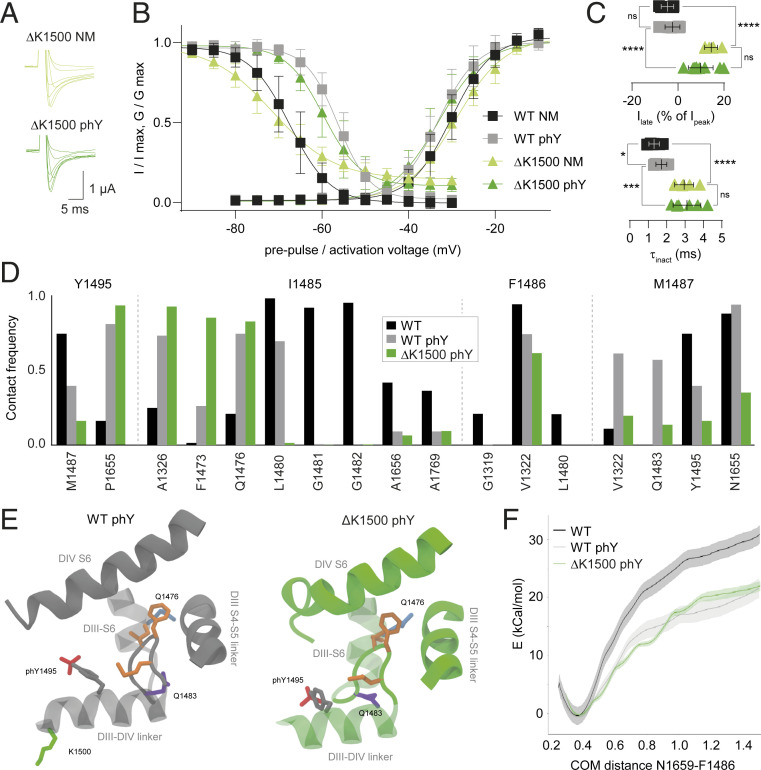
Phosphorylation-induced SSI shift is similar in ΔK1500 and WT. (*A*) Representative current traces for ΔK1500 NM (light green) and ΔK1500 phY (dark green) constructs. (*B*) SSI (*Left*) and activation (*Right*) curves of indicated constructs. (*C*) Late currents (*Upper*) and inactivation rates (*Lower*) for indicated constructs. Data are shown as mean ± SD in *B* and *C*; *n* = 6 to 10; data were compared using unpaired two-tailed Student’s *t* test; ns (not significant) *P* > 0.05, *P ≤ 0.05, ***P ≤ 0.001, and ****P < 0.0001. (*D*) Contact frequency of Y1495 and IFM particle with neighboring residues. (*E*) Conformation of the IFM particle in its binding site after 200 ns of MD simulation. I1485 increases its contacts with DIII-S6 residues. IFM side chains highlighted in orange. (*F*) Free energy profile of IFM unbinding using the distance between the COM of N1659 in the DIV-S5 helix and F1486 in the IFM particle as a reaction coordinate. The binding energy of the IFM particle for the ΔK1500 phY system is similar to that of WT phY.

Next, we set out to assess if other patient mutants in close proximity to Y1495 would show a similar differential modulation by phosphorylation. Specifically, we chose a deletion mutant, ΔK1500, that had previously been associated with LQT3, BS, and conduction system disease (25). To this end, we synthesized P^SYN^ variants containing the ΔK1500 deletion mutation on either the Y1495F (ΔK1500 NM) or the Y1495 phY background (ΔK1500 phY). With both, we observed robust voltage-gated currents (Fig. 3*A*). Interestingly, the midpoints of voltage dependence for activation and SSI were very similar to those observed for the respective WT constructs (compare ΔK1500 NM and WT NM; ΔK1500 phY and WT phY; Fig. 3 *B* or *C* and Table 1). In other words, phosphorylation at position 1495 in the presence of ΔK1500 results in an ∼12-mV right-shift in SSI, which is nearly identical to the shift observed on the WT background. However, the ΔK1500 mutation does reduce the slope of the SSI curve and results in a decreased rate of inactivation and a distinct late current (Fig. 3 *A*–*C* and Table 1), even in the absence of phosphorylation.

Our observation that phosphorylation affects the inactivation properties of ΔK1500 in a manner similar to WT is further corroborated by our computational studies. We find the binding free energy of the IFM particle to its binding site to indeed be indistinguishable from that of WT phY (Fig. 3*F*), despite a slightly altered interaction profile between the IFM particle and its binding site (Fig. 3 *D* and *E*).

Together, we show that the ΔK1500 mutation per se slows the rate of inactivation and results in a substantial late current, thus likely explaining its pathogenicity. However, and in contrast to the Q1476R mutation, the functional effects elicited by phosphorylation at Y1495 are virtually identical to those observed with WT channels.

### Differential Effects of Phosphorylation and Patient Mutations on Na_v_1.5 Pharmacology.

**Fig. 4.**
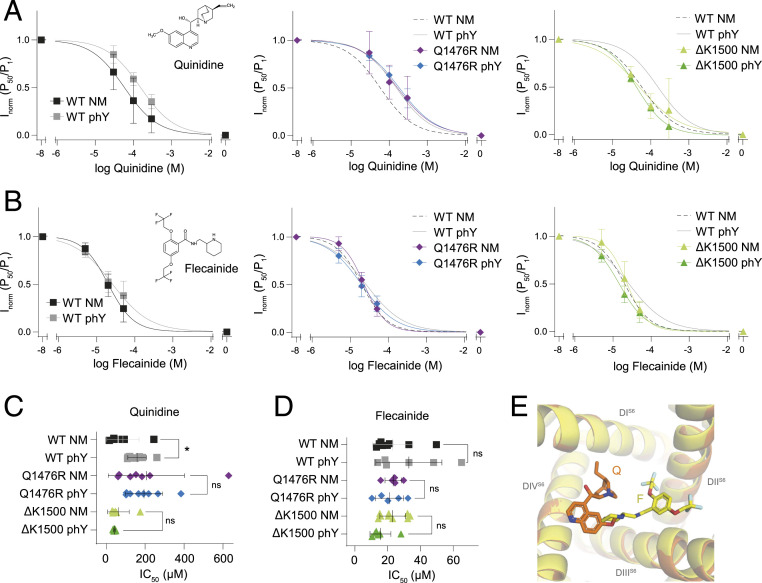
Phosphorylation and disease mutations can affect pharmacological sensitivity of Na_v_1.5. (*A* and *B*) Concentration response curves of WT, Q1476R, and ΔK1500 constructs in response to a 20-Hz pulse train stimulation in presence of AADs quinidine (*A*) or flecainide (*B*), respectively (see structures in left panels). P_50_/P_1_ values were normalized to range from 0 to 1. (*C*) IC_50_ values obtained for quinidine data shown in *A*. The IC_50_ is significantly increased by phosphorylation only in WT but not in Q1476R or ΔK1500 constructs. (*D*) IC_50_ values obtained for flecainide data shown in *B*. IC_50_ values are not significantly altered by phosphorylation in any of the constructs. ns (not significant) *P* ≥ 0.05 and *P < 0.05. Data shown as mean ± SD; *n* = 5 to 9. (*E*) Overlay of flecainide (F) bound to rat Na_v_1.5 (yellow; PDB code: 6UZ0) and quinidine (Q) bound to human Nav1.5 (orange; PDB code: 6LQA).

To test if the above observations have consequences for Na_v_1.5 pharmacology, we next investigated if phosphorylation of Y1495 alone or in combination with the patient mutations would affect the channel’s sensitivity toward clinically used AADs. First, we tested the Class Ia AAD quinidine (Fig. 4*A*), which displays pronounced use-dependent inhibition of Na_v_1.5. Inhibition by quinidine was assessed using a 20 Hz stimulation protocol and yielded a half-maximal inhibitory concentration (IC_50_) of 86 ± 83 μM for WT NM (Fig. 4 *A* and *C* and Table 2). By contrast, phosphorylation of Y1495 increased the IC_50_ to 159 ± 47 μM (WT phY; Fig. 4 *A* and *C* and Table 2). Introduction of the Q1476R mutation led to a further decrease in apparent affinity, but this value was no longer affected by phosphorylation at Y1495 (206 ± 196 μM for Q1476R NM versus of 195 ± 96 μM for Q1476R phY; Fig. 4 *A* and *C* and Table 2). Similarly, on the ΔK1500 mutant background, quinidine inhibition was virtually independent of phosphorylation at Y1495, with IC_50_ values of 63 ± 55 μM (ΔK1500 NM) and 43 ± 5 μM (ΔK1500 phY), respectively (Fig. 4 *A* and *C* and Table 2).

**Table 2.** Pharmacological sensitivity toward clinically relevant AADs

Construct	IC_50_ quinidine (µM)	IC_50_ flecainide (µM)	IC_50_ flecainide (µM)
WT NM	86 ± 83 (6)	23 ± 12 (8)	60 ± 28 (8)
WT phY	159 ± 47 (9)	33 ± 20 (6)	156 ± 68 (6)
Q1476R NM	206 ± 196 (7)	23 ± 5 (5)	80 ± 35 (7)
Q1476R phY	195 ± 96 (8)	21 ± 9 (5)	130 ± 68 (6)
ΔK1500 NM	63 ± 55 (6)	23 ± 8 (9)	57 ± 25 (6)
ΔK1500 phY	43 ± 5 (7)	16 ± 6 (6)	146 ± 67 (7)

IC_50_ for quinidine, flecainide, and ranolazine. Values are provided as mean ± SD; the number of replicates for each value is shown in parentheses.

Next, we turned to inhibition by flecainide, a slightly larger and more hydrophobic AAD (Fig. 4*B*). As expected (34), this Class Ic drug showed generally higher apparent affinity than quinidine (Table 2). However, and in contrast to quinidine, we did not observe a phosphorylation-induced change in apparent affinity on the WT background, and this was also true for the Q1476R and the ΔK1500 mutations (Fig. 4 *B* and *D* and Table 2). Finally, we tested ranolazine, another clinically used sodium channel inhibitor. Like quinidine, ranolazine displayed a reduction in apparent drug affinity on the WT background in response to phosphorylation. This reduction was also observed on the ΔK1500 mutant but not the Q1476R background (*SI Appendix*, Fig. S5).

The above suggests that inhibition of WT Na_v_1.5 by both quinidine and ranolazine is sensitive to phosphorylation at Y1495, while flecainide inhibition is not. Additionally, our data show that the Q1476R mutation abolishes the phosphorylation-induced reduction in apparent affinity for all three tested drugs, while for the ΔK1500 mutation this was only the case for quinidine and flecainide.

## Discussion

In this study, we use a combination of protein semisynthesis and MD simulations to study the effects of phosphorylation on Y1495 on the DIII-DIV linker of Na_v_1.5. Based on this approach, we 1) outline a detailed molecular mechanism for how phosphorylation of Y1495 modulates Na_v_1.5 inactivation, 2) demonstrate that the magnitude of the functional effects caused by this modification can vary substantially when we combine it with a patient mutation, and 3) show that phosphorylation can affect the pharmacological sensitivity of Na_v_1.5 toward clinically relevant AADs.

### Mechanism for How Y1495 Phosphorylation Affects Inactivation in Na_v_1.5.

Although altered fast inactivation (and late currents) alone can be disease-causing (35) (see also Fig. 3 *A*–*C*), it has long been acknowledged that Na_v_1.5 pathogenicity is often associated with changes in the voltage dependence of SSI. For example, the pronounced right-shifted SSI due to phosphorylation of Y1495 is well established from previous in vitro work (18, 33). Yet the precise mechanism of how phosphorylation of Y1495 affects SSI, a process fundamental to Na_v_ function, remained enigmatic. Here, we show that the pronounced (∼11 mV) right-shift in the voltage sensitivity of SSI and the significantly accelerated recovery from inactivation due to phosphorylation of Y1495 is caused by a destabilization of the interactions between the IFM particle and its receptor site, which results in a decrease of binding energy of the IFM particle to its binding site. Our simulations illustrate that phosphorylation causes the IFM particle to be displaced from its receptor site, moving it toward DIII-S6 to accommodate the phosphate group of Y1495 in the binding pocket of IFM. In light of the high degree of sequence conservation in the DIII-DIV linker across mammalian Na_v_ channels (36), we speculate that the mechanism by which Tyr phosphorylation in the DIII-DIV linker affects channel inactivation is likely conserved beyond Na_v_1.5.

It is important to note that Na_v_1.5 SSI likely involves side chains in the C-terminal domain (37–40), possibly including an interaction between the DIII-DIV linker and the channel C terminus (41). However, our present work is unable to directly address the potential role of phosphorylation in this process.

### Mechanistic Insight into Na_v_1.5 Disease-Causing Mutations.

The LQT3 mutant Q1476R had previously been shown to leave the voltage dependence of activation unaffected but to cause a 6.5-mV right-shift in SSI along with a prominent late current (26). Obtained in human embryonic kidney (HEK) 293 cells, these findings are consistent with the notion that Na_v_1.5 LQT syndrome mutants typically result in a gain-of-function phenotype (42). We were therefore surprised to find that in the complete absence of phosphorylation at Y1495 (i.e., on the NM background), the Q1476R mutation itself did not change the voltage dependence of SSI, alter the time course of inactivation, or elicit a late current (Fig. 2). By contrast, introducing the Q1476R mutation on the phY background caused a dramatic (20 mV) right-shift in SSI together with a substantial late current and accelerated recovery from inactivation. The more pronounced SSI shift compared to the one observed in HEK293 cells is not unexpected because in our experiments, 100% of the Q1476R mutant channels are phosphorylated, whereas native phosphorylation levels under baseline conditions are typically lower (19, 20), which would result in a less pronounced right-shift in SSI.

At the molecular level in our MD simulations, Q1476 appears to stabilize the IFM particle in the WT phY system. The Q1476R mutation, however, disrupts this stabilization and causes significant conformational changes leading to weakened IFM particle binding. The functionally observed right-shift in SSI gives rise to a significant window current around −40 mV (43). Together, our data strongly suggest that the pathogenicity of the LQT3 mutant Q1476R does not arise from the Gln-to-Arg exchange per se but rather the combined effect of mutation and phosphorylation.

To validate the above findings and to ascertain that the observed phosphorylation-induced hypershift in SSI was not a nonspecific effect of mutations on the DIII-DIV linker, we also investigated the LQT3/BS mutant ΔK1500. This mutation has previously been shown to cause a minor right-shift in the voltage dependence of activation along with a small left-shift in SSI, slower inactivation, and a late current (25), similar to what had been observed for the classic ΔKPQ deletion mutation, for example (44). Our findings show that the ΔK1500 mutation indeed causes a slower time course of inactivation along with a late current on both the NM and phY backgrounds, although the voltage dependence of activation and inactivation were similar to those observed on the WT background. Most importantly, however, we show that the phosphorylation-induced right-shift in SSI is comparable to that of WT (ΔSSI ∼12 mV for ΔK1500 versus ∼11 mV for WT). This is consistent with our observation that the interaction pattern of the IFM motif with its binding site is no further disrupted than in WT phY, thus underlining that the phosphorylation-induced hypershift in SSI is specific to the Q1476R mutation.

### Na_v_1.5 Pharmacology Can Be Affected by Phosphorylation and Patient Mutations.

Disruptions to inactivation through mutations or other manipulations have previously been shown to weaken binding of local anesthetics to Na_v_ channels (45, 46). This is in line with our finding that quinidine has lower apparent affinity on the WT phY background than on the WT NM background, as the former displays an 11-mV right-shift in SSI. However, on channels with the Q1476R or ΔK1500 mutations, phosphorylation of Y1495 did not lower the apparent affinity toward quinidine despite phosphorylation causing similar (ΔK1500) or even greater (Q1476R) right-shifts in SSI on these channels compared to WT. This suggests that indirect effects on apparent affinity of Class Ia drugs caused by patient mutations can override those caused by altered inactivation.

Interestingly, we find that on the WT background, inhibition by quinidine and ranolazine is sensitive to phosphorylation while that by flecainide is not. At least for quinidine and flecainide, where structural data are available, this difference might be explained by their divergent binding positions and poses (11, 13): While flecainide interacts primarily with S6 of DII and DIII, quinidine interacts most closely with S6 of DIV (Fig. 4*E*). Because the phosphorylated side chain of Y1495 points toward the latter, we speculate that phosphorylation induces an unfavorable conformational change that is most prominent around the quinidine binding site. By contrast, the more drastic conformational consequences of the Q1476R mutation appear to be extensive enough to eliminate any phosphorylation-induced alterations in apparent drug sensitivity.

Although the differences in apparent drug affinity observed by us are comparatively small, our data support the notion that both phosphorylation and disease-causing mutations can impact Na_v_1.5 pharmacology. While disease-causing mutations have been shown previously to affect Na_v_ channel pharmacology (26, 34, 47–49), there is less evidence for phosphorylation-mediated alterations (50). In the future, this may have implications for treatment regimens of patients with known or suspected disease-causing Na_v_1.5 mutations, as some AADs are known to display potentially dangerous proarrhythmic properties in some patient subpopulations (i.e., flecainide and encainide) (51–53).

### Potential Physiological and Clinical Relevance of Phosphorylation-Induced Effects.

To date, most confirmed Na_v_1.5 phosphorylation sites are situated within the DI-DII linker. Although multiple lines of evidence suggest that all intracellular linkers can be phosphorylated (18, 54, 55), mass spectrometry–based work on cardiac and neuronal Na_v_ channels from native tissues has yet to find evidence for phosphorylation in the DIII-DIV linker (20, 56, 57). This is most likely due to the close association of the DIII-DIV linker with the transmembrane helices (11) and its highly basic sequence content (12/53 amino acids are basic), both of which reduce mass spectrometry sequence coverage. By contrast, in vitro studies have provided direct evidence for phosphorylation of Y1495 by Fyn kinase (33, 58) as well as phosphorylation of multiple other Tyr side chains in Na_v_1.5 (59). Additionally, tyrosine kinase inhibitors inhibit sodium currents in rabbit cardiac myocytes by left-shifting SSI (60), in line with findings by us and others (23, 33).

The fact that phosphorylation of Y1495 can impact Na_v_1.5 channel availability (Fig. 1) and that this can be exaggerated by patient mutations (Fig. 2) has potential pathophysiological relevance. This is because Tyr kinases have been shown to be more active under pathological conditions, such as ischemia, reperfusion injury, or cardiac remodeling (61–63). This aspect is further underlined by the observation that β-adrenergic stimulation causes cardiac ion channels, including Na_v_1.5, to undergo increased phosphorylation (20). Here, we demonstrate that Q1476R only causes a severe alteration in channel function when phosphorylated. Together, this may help explain why the clinical phenotype of patients with Na_v_1.5 mutations can vary over time or why individuals with the same mutation are affected to different degrees: in the case of Q1476R, the channel is likely to behave WT-like under basal conditions, but increased phosphorylation levels as a result of metabolic alterations and/or disease states would result in a potentially life-threatening shift in SSI.

Therefore, our data suggest that kinase inhibitors could be a treatment option superior to that of traditional AADs for mutations such as Na_v_1.5 Q1476R (54). Additionally, Na_v_1.5 regulation by calmodulin is at least partly mediated through the DIII-DIV linker (64, 65), thus raising the possibility that the interplay of phosphorylation at Y1495 with patient mutations might extend to Ca^2+^ regulation of Na_v_1.5. Lastly, our work emphasizes that it may be beneficial to tailor future AAD treatment regimens toward the specific genetic background of the patient (49).

### Limitations of Our Study.

To ensure efficient delivery of the synthetic peptides, we used microinjection into *X. laevis* oocytes for our tPTS approach. While this affords unique “on-and-off”–type control over the extent of phosphorylation at a site of interest (0 versus 100%), we cannot assess the extent to which other PTMs might be present in different parts of the protein or if these would differ in a more relevant mammalian expression system. This is important because the functional impact of Na_v_1.5 mutations can be cell-type specific (66–68). Also, Tyr phosphorylation is typically less abundant than that of Ser/Thr (69), so our findings are likely to overestimate the extent to which phosphorylation of Y1495 would affect Na_v_1.5 function in vivo. Similarly, there are minor differences in pK_a_ between the phosphonylation used in the functional experiments and the phosphorylation used for the in silico work (7.5 to 8 versus 6.5) (31, 70).

As a template for our computational work, we used a Nav1.5 structure that was resolved in a presumably inactivated state. This functional assignment is primarily based on the fact that the IFM particle is docked to a site between the DIII S4-S5 linker and DIV S6. However, we note that the construct used for Na_v_1.5 structure determination was heavily engineered, resulting in severe gating shifts and thus prompting us to carefully question this functional assignment. Despite these caveats, our computational data are in remarkable agreement with the functional characterization of the phosphorylation and the patient mutations, leading us to confidently propose a mechanistic model for the effects caused by these modifications.

### Conclusion.

Our study highlights the power of semisynthetic approaches to decipher complex biophysical interactions. Specifically, the data call for caution when interpreting the apparent functional effects of potentially pathogenic mutations because, at least in some cases, the functional consequences of PTMs may outweigh those caused by mutations. This adds another layer of complexity to the investigation and interpretation of Na_v_1.5 patient mutations because PTM levels are subject to variation. Given the enormous (>500) number of disease-associated mutations in Na_v_1.5 alone (1), our findings are likely to be relevant beyond the confines of this study. Our work further motivates the investigation of thus far unexplored cross-talk between PTMs and pathological mutations in other proteins, particularly because PTM-mediated effects may not be confined to spatially close regions of the protein. Finally, the results of our study provide a starting point for understanding how Na_v_1.5 mutations can impact the patient-specific pharmacological sensitivity that is observed clinically.

## Methods

### Molecular Biology and Chemicals.

Gene constructs were generated and used as described in ref. 23; see supplementary information for sequence details. Standard site-directed mutagenesis was performed using PCR, and deletions were created using the Q5 site-directed mutagenesis kit (Thermo Fisher Scientific). The complementary DNA was linearized and transcribed into complementary RNA (cRNA) for oocyte microinjection using the Ambion mMESSAGE mMACHINE T7 Transcription Kit (Thermo Fisher Scientific).

All chemicals were purchased from Sigma-Aldrich, Iris Biotech, Rapp polymere, Combi-Blocks and Chem-Impex unless specified otherwise. AADs were purchased from Sigma-Aldrich (quinidine sulfate salt dihydrate, catalog number: Q0875; mexiletine hydrochloride, catalog number: M2727; flecainide acetate salt, catalog number: F6777; ranolazine dihydrochloride, catalog number R6152) and stored according to product specification. Substances were dissolved in ND96 solution (see *Two-Electrode Voltage Clamp Recordings* section below for composition) and diluted to the specified concentrations, and the pH value was adjusted to 7.4. The prepared solutions were then stored at room temperature and used for no longer than 20 d.

### Peptide Synthesis.

Peptides for Na_v_1.5 splicing were synthesized by solid-phase peptide synthesis as previously described in detail (23). Briefly, P^SYN^ variants were synthesized by ligating three shorter fragments: the N-terminal intein half (Int^C^-A), the sequence derived from the Na_v_1.5 ion channel, and the C-terminal intein half (Int^N^-B), with the Na_v_1.5 ion channel sequence being the only variable one. Regarding the ligation strategy adopted, the three fragments were ligated in a “one-pot” fashion and in a C-to-N direction, exploiting the Thz masking group as previously established (71). Note that the WT NM and phY WT peptides also contained the K1479R mutation (23).

Unless otherwise stated, the amino acids used for solid-phase peptide synthesis were Fmoc-Ala-OH, Fmoc-Cys(Trt)-OH, Fmoc-Phe-OH, Fmoc-Gly-OH, Fmoc-Ile-OH, Fmoc-Lys(Boc)-OH, Fmoc-Leu-OH, Fmoc-Pro-OH, Fmoc-His(Trt)-OH, Fmoc-Asn(Trt)-OH, Fmoc-Gln(Trt)-OH, Fmoc-Arg(Pbf)-OH, Fmoc-Ser(*t*Bu)-OH, Fmoc-Thr(*t*Bu)-OH, Fmoc-Tyr(*t*Bu)-OH, Fmoc-Asp(*t*Bu)-OH, Fmoc-Glu(*t*Bu)-OH, Fmoc-Met-OH, Fmoc-Val-OH, and Boc-Thz-OH.

Automated peptide synthesis was carried out on a Biotage Syro Wave peptide synthesizer using standard Fmoc/tBu SPPS chemistry as previously reported (23). Fmoc deprotection was performed by treatment with piperidine–*N,N*-dimethylformamide (DMF)–formic acid (25:75:0.95, vol/vol/vol), 3 + 12 min. Coupling reactions were performed as double couplings using Fmoc-Xaa-OH (6.0 equivalent to the resin loading), *O*-(6-Chlorobenzotriazol-1-yl)-*N,N,N’,N’*-tetramethyluronium hexatuorophosphate (HCTU, 6.0 equivalent), and *N,N*-diisopropylethylamine (*i*-Pr_2_NEt; 12 equivalent) for 40 min for each coupling. All reagents and amino acids used during SPPS were dissolved in *N,N*-dimethylformamide except *i*-Pr_2_NEt, which was dissolved in *N*-methyl-2-pyrrolidinone. Coupling reactions for nonstandard Fmoc-protected amino acids were performed as outlined for each peptide (*SI Appendix*).

Thioesterification of peptides was performed as described (23, 72) and upon cleavage of protected peptides from resin with 1,1,1,3,3,3-hexafluoro-2-propanol–dichloromethane (20:80, vol/vol). General deprotection of the peptides was performed with a mixture of trifluoroacetic acid (TFA)–2,2´-(ethylenedioxy)diethanethiol (DODT)–triisopropylsilane (94:3.3:2.7, vol/vol/v) for 60 to 90 min. Upon full deprotection (monitored by matrix-assisted laser desorption/ionization–time of flight), the reaction mixture was concentrated under a stream of nitrogen and the crude peptide was precipitated by addition of cold diethyl ether. The solid was spun down and subsequently washed with cold diethyl ether (2×). In case of partial oxidation of methionine residues, this could be reversed by incubating the peptide in a mixture of DODT (0.2 M) and bromotrimethylsilane (0.1 M) in TFA for 20 min (23, 73). The peptide was then precipitated from diethyl ether before proceeding with preparative high-performance liquid chromatography purification.

### Expression in *X. laevis* Oocytes.

Stage V-VI oocytes from *X. laevis* oocytes (prepared as described previously in ref. 23) were injected with cRNAs and incubated at 18 °C in OR-3 (Oocyte Ringer 3) solution (50% Leibovitz’s Medium, 1 mM L-glutamine, 250 mg/L gentamicin, 15 mM HEPES, pH 7.6) for up to 3 d. The lyophilized synthetic peptides were dissolved in Milli-Q H_2_O to a concentration of 500 to 750 µM, and 9 to 14 nL of solubilized peptide was injected into oocytes preinjected with cRNA using a Nanoliter 2010 micromanipulator (World Precision Instruments). Typically, synthetic peptides were injected about 24 h after cRNAs injection. Recordings were conducted 12 to 20 h after injection of synthetic peptides.

Note that experimental uncertainties associated with the injection of small amounts of synthetic peptides do not allow us to draw conclusions on expression levels (e.g., to directly compare current sizes between WT and mutant channel variants).

### Two-Electrode Voltage Clamp Recordings.

Voltage-dependent currents were recorded with a two-electrode voltage clamp using an OC-725C voltage clamp amplifier (Warner Instruments). Oocytes were constantly perfused with ND96 solution (in mM: 96 NaCl, 2 KCl, 1 MgCl_2_, 1.8 CaCl_2_/BaCl_2_, 5 HEPES, pH 7.4) during recordings. For pharmacological testing, solutions containing different AADs in ND96 were used. Glass microelectrodes with resistances between 0.1 and 1 MΩ were backfilled with 3 M KCl. To determine values for half-maximal activation, oocytes were held at −100 mV and sodium currents were elicited by voltage steps from −80 mV to +40 mV (in 5-mV increments). SSI curves were determined by applying a 500-ms prepulse from −100 mV to −20 mV (in +5-mV increments) followed by a 25-ms test pulse to −20 mV.

### MD Simulations.

#### Model building.

First, the Na_v_1.5 channel (Protein Data Bank [PDB] ID 6UZ3) (11) was embedded in a homogenous lipid bilayer consisting of 400 1-palmitoyl-2-oleoyl-sn-glycero-3-phosphocholine (POPC) molecules using the CHARMM-GUI Membrane Builder (74). Four different systems were prepared: 1) WT Nav1.5, 2) Na_v_1.5 with Y1495 phosphorylated, 3) Na_v_1.5 with Y1495 phosphorylated and Q1476R mutation, and 4) Na_v_1.5 with Y1495 phosphorylated and ΔK1500 mutation. Phosphorylation and mutation were performed using the CHARMM-GUI Membrane Builder. The system was hydrated by adding two ∼25 Å layers of water to both sides of the membrane. Lastly, the system was ionized with 150 mM NaCl.

#### Simulations.

The CHARMM36 force field was used to describe interactions between protein (75), lipid (76), and ions, and the transferable intermolecular potential 3-point (TIP3P) model was used to describe the water particles (77). The systems were minimized for 5,000 steps using steepest descent and equilibrated with constant number of particles, pressure, and temperature (NPT) for at least 36 ns for all four systems, during which the position restraints were gradually released according to the default CHARMM-GUI protocol (78). During equilibration, a time step of 2 fs was used; pressure was maintained at 1 bar through Berendsen pressure coupling; temperature was maintained at 300 K through Berendsen temperature coupling (79) with the protein, membrane, and solvent coupled; and the LINCS algorithm (80) was used to constrain the bonds involving hydrogen atoms. For long range interactions, periodic boundary conditions and particle mesh Ewald were used (81). For short range interactions, a cutoff of 12 Å was used. Finally, unrestrained production simulations were run for 200 ns for each of the systems using Parrinello-Rahman pressure coupling (82) and Nosé-Hoover temperature coupling (83). Simulations were performed using GROMACS 2019.3 (84, 85).

#### Umbrella sampling.

To calculate the free energy profile of binding of IFM to its binding site, umbrella sampling was employed using the distance between the COM of F1486 and the COM of N1659 as a reaction coordinate. Each umbrella sampling window was 1 Å wide, and the position of the IFM particle was restrained by applying a harmonic potential on the reaction coordinate. A brief 100-ps-long NPT equilibration was conducted with 1 atm pressure maintained by Berendsen pressure coupling and 300 K, controlled by Berendsen thermostat. This was followed by 10 ns of production simulation in each window. The N1659 residue (located in the IFM particle binding site) was restrained throughout the simulation with a restraining potential force constant of 1,000 kJ ⋅ mol^−1^ ⋅ nm^−2^. The weighted histogram analysis method (WHAM) (86) in GROMACS (gmx wham) was used to combine data from all the umbrella sampling windows to compute the free energy profile.

#### Contacts.

Contacts are defined when the distance between the Cβ atoms of pairs of residues are below 6.7 Å. Those were calculated using MD-TASK (87).

## Supplementary Material

Supplementary File

## Data Availability

Electrophysiology data have been deposited in Zenodo (https://doi.org/10.5281/zenodo.4778147) (88). Molecular modeling and MD simulations files have been deposited on the Open Science Framework (OSF) repository (https://osf.io/hsyx4/) (89).
